# D-dimer surge and coagulation disorders in COVID-19 related pneumonia patients with cardiac injury: A case series

**DOI:** 10.1097/MD.0000000000021513

**Published:** 2020-07-31

**Authors:** Xingtong Wang, Beibei Du, Jia Li, Shunan Wang, Xiuxiu Wang, MengYuan Guo, Bo Yang, Daoyuan Si, Ou Bai

**Affiliations:** a Department of Hematology, The First Hospital of Jilin University, Jilin Provincial Hematology Research Institute, National Key Discipline; b Department of Cardiology, China-Japan Union Hospital of Jilin University, Jilin Provincial Cardiovascular Research Institute, Changchun; c Institute of Organ Transplantation, Tongji Hospital, Tongji Medical College, Huazhong University of Science and Technology, Wuhan, China.

**Keywords:** cardiac injury, case series, coagulation disorders, COVID-19, D-dimer surge

## Abstract

**Background:**

Cardiac injury and coagulation disorders have been two increasing concerns in the management of patients with severe coronavirus disease (COVID-19). Coagulation disorders in COVID-19 patients with cardiac injury have not been characterized.

**Methods:**

We analyzed the data of five COVID-19 patients with cardiac injury who had D-dimer surge (defined as a rapid increase in the D-dimer level in 72 h, from <5–21 μg/mL) during hospitalization, which were extracted from a registered retrospective study (ChiCTR2000031301). Clinical data and data on changes in coagulation parameters were collected, verified, and characterized.

**Results:**

Among these five patients, four had pre-existing cardiovascular or cerebrovascular diseases. D-dimer surge was accompanied with prolonged prothrombin time (PT) and reduced platelet count (PLT) and fibrinogen level. Three patients had an ISTH DIC score of 5 and met the criteria for overt DIC. All five patients needed invasive ventilation support and were incubated 0 to 6 days after the first D-dimer upper reference limit (URL) was reached. All five patients died within 10 days after the first D-dimer URL was reached. All five patients had observed D-dimer URL results 1 to 3 days before death.

**Conclusion:**

D-dimer surge in COVID-19 patients with cardiac injury surely leads to worse in-hospital outcome. D-dimer surge and concomitant DIC can be the leading causes of in-hospital death. Pre-existing cardiovascular or cerebrovascular diseases might pose a higher risk for developing these coagulation disorders. These findings can serve as hypothesis generating and need further clinical trials to confirm.

## 1 Introduction

Coronavirus disease (COVID-19) patients have a 7% to 20% incidence of cardiac injury (indicated by elevated cardiac troponin I level [cTnI]).^[1–3]^ COVID-19-related pneumonia patients with cardiac injury have an extremely high mortality (51%), around 10 times higher than patients without cardiac injury.^[1]^ Though cardiac injury was generally speculated as being myocarditis, no mechanism for how cardiac injury increased the mortality has been clarified yet.

Existing data also show that patients with severe COVID-19 often have coagulation dysfunction and an elevated D-dimer level (46.4%).^[4,5]^ Abnormal coagulation function further increased the difficulty of treatment and increased mortality (11.5%).^[4]^ Whether COVID-19 patients with cardiac injury have coagulation dysfunction and how it affects the clinical course and prognosis have not yet been reported. Here, we report a case series of five COVID-19 patients with cardiac injury and D-dimer surge and describe their clinical characteristics.

## 2 Materials and methods

This case series was from a retrospective study of 170 COVID-19 patients with cardiac injury at admission (defined as an admission cTnI level >99th percentile of the upper reference limit [URL] [26.2 pg/mL]). This study was approved by the research ethics boards of China-Japan Union Hospital of Jilin University (approval number 2020032619), Tongji Hospital, Huazhong University of Science and Technology (approval number TJ-IRB20200345) separately, which was also registered in the Chinese Clinical Trial Registry (ChiCTR2000031301). Informed consents from all the five patients were obtained for the publication of this study and any accompanying images.

When analyzing the data, we noted that there were many patients with D-dimer surge during the hospitalization. D-dimer levels were tested according to the discretion of the COVID-19 team. Five patients among them had experienced D-dimer surge (pre-defined as a rapid increase in the D-dimer level, from a low level to 21 μg/mL [URL]) within 72 h. The baseline D-dimer levels of all five patients were <5 μg/mL (median [range], 2.08 [0.63–4.4] μg/mL, normal range < 0.5 μg/mL). Thus, we defined D-dimer surge as an increase in the D-dimer level from <5 μg/mL to 21 μg/mL in 72 h. These five patients’ clinical and coagulation data were extracted and analyzed.

## 3 Results

### 3.1 Patient demographics

According to the triage policy, all five patients were transferred to a regional care center, Tongji Hospital, from nearby hospitals or clinics once they were diagnosed with CT-proven COVID-19-related pneumonia.

All five patients were male and ≥60 years old (range, 60–82 years). All had typical symptoms of severe acute respiratory syndrome coronavirus 2 (SARS-CoV-2) infection on admission, such as fever (median [range], 38.5 [38.1–40.0] °C), dry cough, and dyspnea.

**Figure 1:**
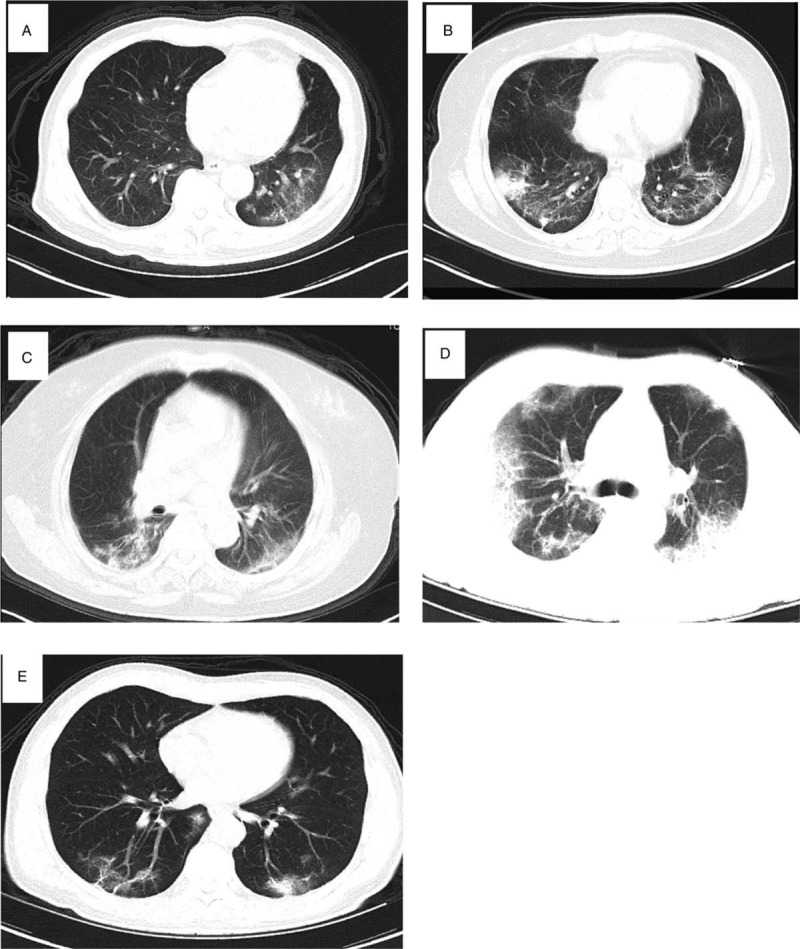
Representative pulmonary CT images of COVID-19-related pneumonia. Panels A–E represent pulmonary CT results of patients 1 to 5.

The diagnosis was made according to WHO interim guidelines,^[6]^ based on the typical symptoms of SARS-CoV-2 infection, prior contact history, and positive SARS-CoV-2 nucleotide acid test results. COVID-19 related pneumonia was diagnosed according to typical patches of “ground glass” changes on CT scans (Fig. 1A–E).

**Figure 2:**
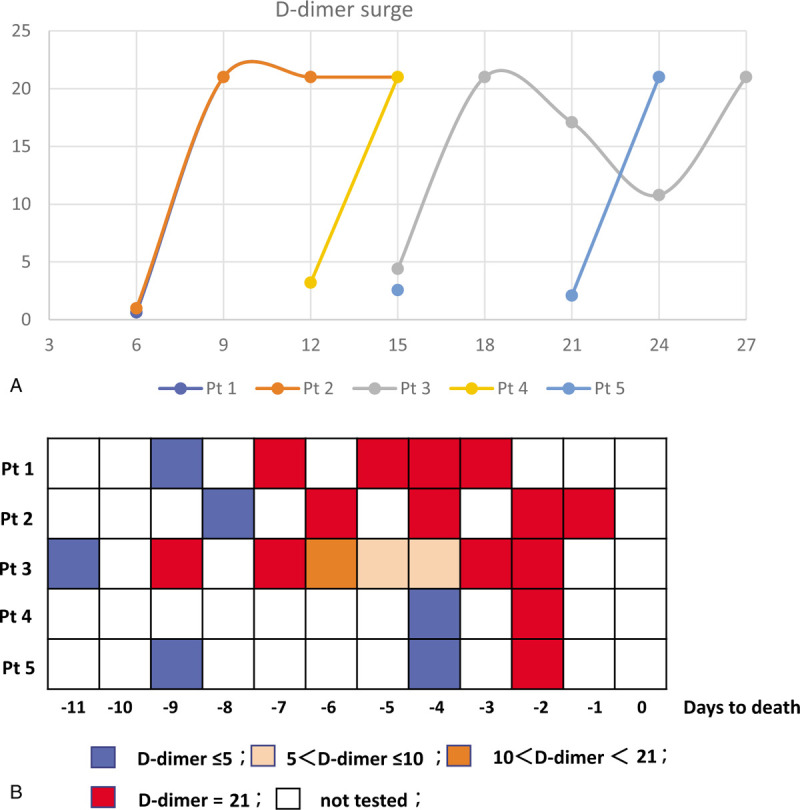
D-dimer surge and its relation with in-hospital death in COVID-19 patients with cardiac injury. Panel A: D-dimer surge in 5 patients. Time is calculated since the onset of illness. Please note that the changes in the curves of D-dimer levels in patients 1 and 2 are highly coincident. Panel B: D-dimer level, D-dimer surge, and in-hospital death. Time is calculated since death. Different D-dimer levels are marked with different colors.

All five patients had cardiac injury at the time of admission, defined as an elevated cTnI level (median [range], 653.2 [59–6788.7] pg/mL) (Table 1). In all these patients with cardiac injury at admission, D-dimer surge was observed during hospitalization (Fig. 2A and B). The baseline D-dimer level was 2.08 [0.63–4.4] μg/mL (median [range]).

**Table 1:**
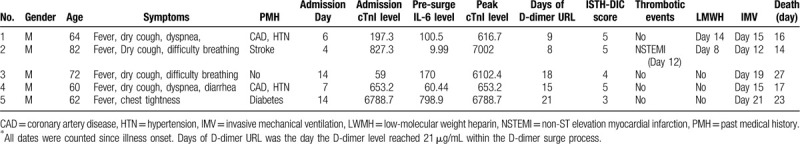
Demographics of the 5 patients with D-dimer surge.

Four of the five patients had pre-existing cardiovascular or cerebrovascular diseases (2 had coronary artery diseases [CAD] and hypertension, 1 had CAD and diabetes, and 1 had stroke). The cTnI level (range, 59-6788.7 pg/mL), peak cTnI level (range, 616.7–6788.7 pg/mL), and IL-6 level at admission, prior to D-dimer surge (range, 9.99–798.9 pg/mL), were all elevated, but varied vastly (Table 1).

### 3.2 D-dimer surge and disseminated intravascular coagulation (DIC)

All patients had D-dimer surge during hospitalization; the median time for the D-dimer level to reach its first peak was day 15 (range, [8–21]) since symptom onset. Before and after this D-dimer peak, coagulation parameters were monitored according to physicians’ decisions.

**Figure 3:**
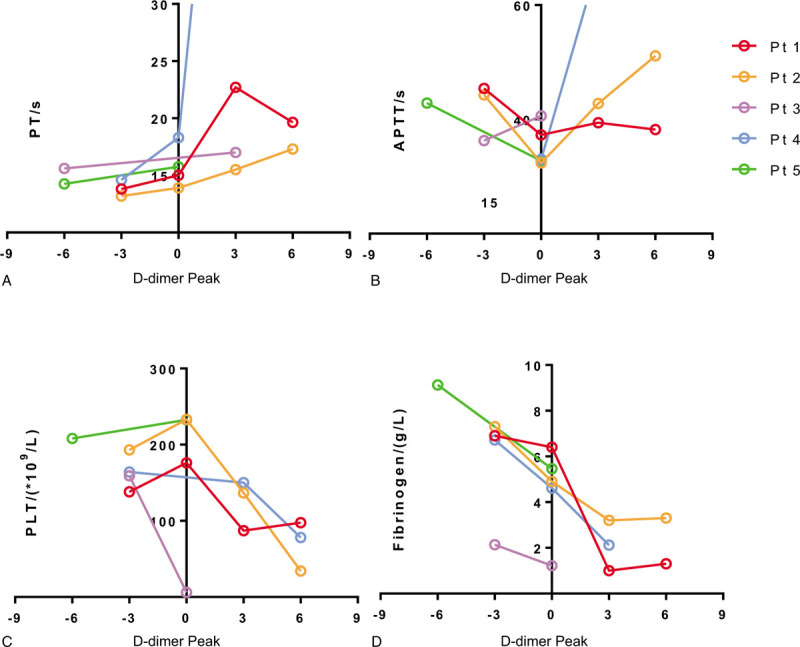
Concomitant coagulation disorders during D-dimer surge. (A–D) Panel plot of concomitant PT, APTT, PLT count, and fibrinogen changes during and shortly after D-dimer surge. APTT = activated partial thromboplastin time, PLT = platelet, PT = prothrombin time.

Panel data of the five patients showed temporal changes in prothrombin time (PT), activated partial thromboplastin time (APTT), PLT count, and fibrinogen level. PT showed a trend to prolong, while PLT count and fibrinogen level showed decreasing trends during and soon after the D-dimer surge. APTT showed no consistent trend of change (Fig. 3A–D). The International Society of Thrombosis and Haemostasis (ISTH) DIC scores were assessed; three patients scored 5 (overt DIC), one scored 4 (non-overt DIC), one scored 3 (non-overt DIC). However, in-hospital thrombotic event was only observed in one patient with non-ST elevation myocardial infarction (NSTEMI), which was verified with inferior wall and anterior wall T wave inversions (Table 1).

### 3.3 D-dimer surge, treatment, and in-hospital outcome

All patients were administered antiviral (Arbidol in 4 patients, Oseltamivir in 2 patients, Ganciclovir in 1 patient) and antibiotic (Moxifloxacin in 3 patients, Cefoperazone in 2 patients, Amoxicillin in 1 patients) treatments. Two patients (patients 1 and 2) were administered low-molecular weight heparin (LMWH, 4000 IU/day, sc) according to physician's discrepancies. All five patients were allowed a 0 to 6 day incubation period after the first D-dimer URL was reached. All five patients died within 10 days after the first D-dimer URL was reached. In all patients, the D-dimer URL results were observed at the most 3 days before death (Fig. 2B). The direct causes of death in these five patients were multiple organ dysfunction syndrome (MODS) (patients 1, 3, and 4), acute respiratory distress syndrome (ARDS) (patient 5), and ARDS and ventricular fibrillation (patient 2).

## 4 Discussion

Herein, we have first reported the characterizations of D-dimer surge and related coagulation disorders in five COVID-19 patients with cardiac injury. These patients had a poor prognosis as theoretically expected. D-dimer surge had a high tendency of meeting the requirement for DIC. Pre-existing cardiovascular and cerebrovascular diseases might be risk factors for this coagulation disorder. Changes in these biomarkers may be valuable for risk stratification and guiding an active surveillance strategy for COVID-19 patient management.^[7]^

Cardiac injury and coagulation disorders are both common in patients with severe COVID-19.^[8–10]^ Whether COVID-19 patient with cardiac injury have coagulation disorders and how they may affect the clinical course are unknown. In our case series, the patients with cardiac injury did show severe coagulation derangements, which closely correlated with clinical deterioration, such as invasive mechanical ventilation and in-hospital death. D-dimer surge and related coagulation disorders (such as DIC) can be a direct cause of MODS and in-hospital death in these COVID-19 patients with cardiac injury.

Patients with severe COVID-19 who eventually died exhibited an elevated D-dimer level and an underlying pro-thrombotic state. In a study of 183 COVID-19 patients, elevated D-dimer and fibrin degradation products were observed in the non-survivors (11%); 15 non-survivors met the ISTH criteria for overt DIC, whereas only one survivor developed overt DIC.^[4]^ In our study also, three of the five patients with D-dimer surge met the ISTH criteria for overt DIC, and one patient developed NSTEMI.

COVID-19-related coagulopathy can present different coagulation changes with different stages of the patients.^[9]^ Complex mechanisms such as cytokine storm, sepsis/severe infection, and organ injury may all be involved in these coagulation disorders.^[9]^ An early coagulation activation stage was characterized by elevated D-dimer levels, fibrinogen, PT, and so on. Later stage patients may experience uncontrollable coagulation and develop DIC (as per the ISTH guideline), which features as thrombocytopenia, D-dimer surge, prolonged PT and APTT, and rapid decrease in the fibrinogen level.^[4,5,9]^ The five patients showed a fulminant coagulation process; however, whether COVID-19 patients with cardiac injury easily develop this severe coagulation disorder is not known.

COVID-19-related thrombotic risk and ischemic injury are regarded to be more related to microvascular embolism^[11,12]^ than macrovascular thromboembolism. Acute pulmonary embolism and deep venous thrombosis were reported in patients with SARS-CoV-2 infection.^[13,14]^ Due to limited investigations, CT angiography tests, and pathological evidences, the related macrovascular embolisms remain underestimated.

Among COVID-19 patients with cardiac injury, around 30% to 50% had pre-existing cardiovascular or cerebrovascular diseases.^[1,2,15]^ Endothelial injury and dysfunction are generally accepted as the fundamental pathological changes during cardiovascular or cerebrovascular diseases.^[16]^ In contrast, vascular endothelial injury is one of the possible mechanisms of the increased risk of coagulation disorder and DIC^[17]^ in COVID-19 patients. Among our five patients with D-dimer surge, four had pre-existing cardiovascular or cerebrovascular diseases, which might imply that endothelial injury or dysfunction might play a role in coagulation disorders in COVID-19 with cardiac injury. However, this still needs prospective or large retrospective studies to confirm.

Heparin treatment has been shown to decrease mortality in COVID-19 patients with coagulation disorders.^[5,18]^ Among our five patients, two had been treated with heparin (4000 IU, sc, qd) but whether heparin treatment can improve prognosis in COVID-19 patients with cardiac injury cannot be concluded with these limited data.

## 5 Limitations

As a result of the limited patients included, our findings should be interpreted as hypothesis generating and provide supporting evidence of complex pathophysiological status in COVID-19 patients with cardiac injury. Consequently, most data were descriptive to ensure the accuracy, prospective study should be implemented to verify the coagulation status disorders in COVID-19 patients with cardiac injury.

## 6 Conclusions

D-dimer surge during hospitalization is a marker for worse prognosis in COVID-19 patients with cardiac injury. D-dimer surge and concomitant coagulation disorders might be direct reasons for in-hospital deaths. Pre-existing cardiovascular or cerebrovascular diseases might pose a higher risk for developing these coagulation disorders. These findings can serve as hypothesis generating and need further clinical trials to confirm.

## Author contributions

XW, BD, and DS were the patient's physicians. BD, and DS applied for the IRB approval, and registered the retrospective study, JL, BY, XW and SW extracted the data. XW and MG analyzed the data and prepared the figures, XW, BD, and OB reviewed the literature and contributed to manuscript drafting. XW, JL, and OB were responsible for the revision of the manuscript for important intellectual content. All authors issued final approval for the version to be submitted.
